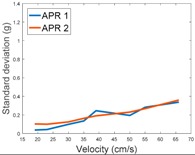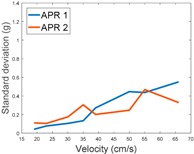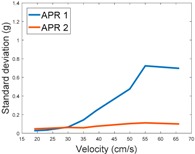# Measurement of Vibrations in Two Tower-Typed Assistant Personal Robot Implementations with and without a Passive Suspension System

**DOI:** 10.3390/s17051122

**Published:** 2017-05-14

**Authors:** Javier Moreno, Eduard Clotet, Marcel Tresanchez, Dani Martínez, Jordi Casanovas, Jordi Palacín

**Affiliations:** 1 Department of Computer Science and Industrial Engineering, University of Lleida, 25001 Lleida, Spain; jmoreno@diei.udl.cat (J.M.); eclotet@diei.udl.cat (E.C.); mtresanchez@diei.udl.cat (M.T.); dmartinez@diei.udl.cat (D.M.); 2 Department of Chemistry, University of Lleida, 25001 Lleida, Spain; jcasanovas@quimica.udl.cat

**Keywords:** holonomic robot, omnidirectional wheels, spring-based suspension system, vibration, tower-typed mobile robots, Assistant Personal Robot

## Abstract

This paper presents the vibration pattern measurement of two tower-typed holonomic mobile robot prototypes: one based on a rigid mechanical structure, and the other including a passive suspension system. Specific to the tower-typed mobile robots is that the vibrations that originate in the lower part of the structure are transmitted and amplified to the higher areas of the tower, causing an unpleasant visual effect and mechanical stress. This paper assesses the use of a suspension system aimed at minimizing the generation and propagation of vibrations in the upper part of the tower-typed holonomic robots. The two robots analyzed were equipped with onboard accelerometers to register the acceleration over the *X*, *Y*, and *Z* axes in different locations and at different velocities. In all the experiments, the amplitude of the vibrations showed a typical Gaussian pattern which has been modeled with the value of the standard deviation. The results have shown that the measured vibrations in the head of the mobile robots, including a passive suspension system, were reduced by a factor of 16.

## 1. Introduction

The use of mobile robots is gaining popularity as an alternative way to offer reliable and more efficient solutions to problems faced not only in industries [1,2,3] but also in domestic environments [4]. Providing appropriate assistance and care to elderly people has become one of the main concerns in developed countries. This problem has already been highlighted by organizations such as the United Nations [5] and the World Health Organization [6], whose studies on the evolution of the world population postulate that the percentage of elderly people (aged 60 or more) will rise from 12% to 21% during the next 35 years, as a result of the clear increase of human life expectancy. In this context, the use of robots in domestic environments is a way of offering proper assistance to people of advanced years due to the evolution of robotics and electronic health systems.

The motion system of assistive robots must be able to maneuver in unstructured and limited spaces without interfering with the inhabitant’s lifestyle. This is a disadvantage for mobile robots equipped with conventional two-independent driving wheels, as their mobility is restricted to one directional movement due to the motion system only providing two degrees of freedom (DOF). As a result, mobile robots equipped with classical steering mobility systems are unable to perform lateral displacements. To overcome these limitations, it is essential to develop omnidirectional mobile robots (OMR), where the term omnidirectional describes the ability of a motion system to change the direction of movement without having to perform any intermediate rotation. As the Assistant Personal Robot (APR) [7] is equipped with three omnidirectional wheels shifted 120° (kiwi drive), it offers a 3-DOF motion system over a two dimensional plane [8]. This allows the robot to move in any angle or direction [9], as well as perform arbitrary movements in arbitrary directions without changing its orientation.

The primary components involved in creating an OMR are the omnidirectional wheels. There are several types of omnidirectional wheels which are based on the same operating principle, providing traction in the direction normal to the motor axis while using the inner passive rollers placed along the periphery of the main wheel which can, as well, slide in the direction of the motor axis.

**Figure 1:**
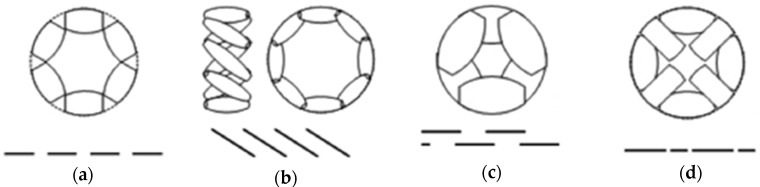
Types of omnidirectional wheels and their traces: (**a**) multiple passive rollers (or inner passive wheels) in which the axes are positioned tangent to the main wheel circumference; (**b**) with the rollers arranged in an overlapping way where the contact between the wheels and the ground is continuous; (**c**) based on two overlapping parallel wheels; (**d**) based on using alternating passive rollers with different size and shape.

Figure 1 shows different types of omnidirectional wheels and their traces. Figure 1a shows a wheel (universal wheel) designed with multiple passive rollers (or inner passive wheels) where the axes are at a tangent with the main wheel circumference. This construction cannot avoid the existence of discontinuities in the trace; therefore, it provides irregular contact with the surface due to the presence of gaps between the successive rollers producing vibrations in the robot. Some solutions are proposed to minimize those effects in these types of wheels, such as reducing the size of the gap between the passive rollers [10]. Figure 1b shows the Mecanum [11,12], invented in 1973 by Bengt Ilon, an engineer working for the Swedish company Macanum AB. This wheel is based on the use of overlapping rollers providing continuous contact between the wheel and the ground. The design and adjustment of this type of wheel’s parameters allow for a remarkable decrease of the vibrations [13]. These wheels are often positioned in pairs over the same axle but in opposite orientations to a four-wheel structure. The drawback of these wheels is the generation of horizontal vibrations due to the parasite torque generated as a result of the contact points moving along a parallel line to the wheel shaft. Figure 1c shows the double wheel concept based on the use of two overlapping parallel wheels. In this case, the contact between the assembled wheel and the ground is continuous; however, this design still generates a significant horizontal vibration originated by the gaps between the rotating inner wheels [14]. Finally, in the design shown in Figure 1d the contact points are aligned in order to reduce the horizontal vibration while using alternating passive rollers of different sizes and shapes in order to minimize the gap between them, thus causing a slight vertical vibration. As with Macanum wheels, there are jobs where vibrations are analyzed according to the design parameters [15].

The common point of the omnidirectional wheels (Figure 1) are the vibrations generated due to the inner trace wheel discontinuities. In the case of the tower-typed mobile robots, these vibrations can cause visible oscillations at the top of the mobile robot. For example, the proposal of [16] analyzes this problem in the case of two-wheeled mobile robots, testing a vibration minimization technique based on using different acceleration and deceleration velocity profiles along with the effects of using soft and hard wheels instead of using a dedicated suspension system. The occurrence of vibrations is frequently used for reconnaissance of the terrain [17,18,19,20], or even power generation [21], but is often a problem for the safety of the components and for their correct operation and comfort [22,23,24,25,26]. The vibrations appearing at the head of the mobile robot cause mechanical stress, cracks [27], and also an undesired visual effect during the displacements. 

This paper proposes the measurement of the vibrations originating in two tower-typed APR mobile robot prototypes: APR-1 and APR-2. The second prototype has been improved by including a suspension system mounted at the holonomic base, which is equipped with three omnidirectional wheels based on the alternation of passive rollers (previously exemplified in Figure 1d). The vibration patterns of the two mobile robots have been obtained by measuring the accelerations at one wheel, chassis, and at the top of the structure.

## 2. Materials and Methods 

The materials used in this paper are two prototypes of the mobile robot APR. The mechanical structure of the first APR-1 prototype was described in [7] and the second improved APR-2 prototype was described in [28]. This paper is focused on the measurement of the vibration patterns found in both prototypes by using multiple linear three-axis accelerometer devices in order to gather the vibration patterns from different locations on the mobile robots and evaluate the improvements achieved. 

### 2.1. Mobile Robot Prototypes

**Figure 2:**
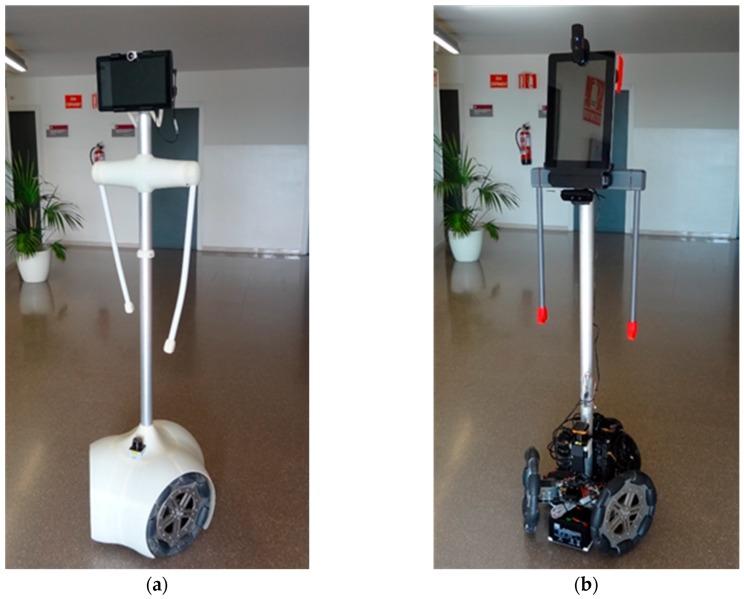
Images of the two mobile robot prototypes analyzed; (**a**) APR-1, (**b**) APR-2.

The experimental part of this paper has been carried out using two APR prototypes (Figure 2); both devices are tower-typed omnidirectional mobile robots with three omnidirectional wheels shifted 120°. The APR-1 [7] was the first APR prototype implemented (Figure 2a) and was built on a rigid structure with a motion system, with the wheels and a tower directly attached. The APR-2 was the second improved prototype implementation (Figure 2b) and it includes a passive suspension system implemented at the base of the robot.

Humans are the inspiration for the physical design of the APR, which resembles them; it is designed to navigate through environments planned to ease their mobility and it is also capable of moving its head and arms. The mobile robot design lacks, on purpose, sharp edges or projecting parts for safety reasons and to facilitate its use in domestic unstructured environments. The structure of the APR is also designed to maintain its reliability as a multipurpose mobile platform; therefore, the main body of the robot is a single thin aluminum tube, on which various measuring devices can be easily clamped on.

The inner mechanical structure of the APR was made with a combination of stainless steel and aluminum parts to guarantee its durability and resistance. All of the heavy elements are located at the base of the robot, lowering its center of mass almost to ground level, and also reducing vibrations at the top of the robot. In both prototypes, the structure of the APR is divided in two parts, the holonomic motion system and the thin body or tower.

The mobile robot has a circular base section which supports the holonomic motion system composed of three omnidirectional wheels, the batteries, the Light Detection and Ranging (LIDAR) device, and the main electronic boards. The inner design of the base is covered with a low-cost 3D printed thermoplastic polymer, Acrylonitrile Butadiene Styrene (ABS), which provides flexible protection that will contribute to absorbing part of the energy of impacts in the case of collision. The circular design minimizes the probability of getting accidentally hooked on furniture objects such as mats, curtains, or clothing, and plays a crucial role when operating in tight indoor spaces, simplifying the mobile robot tele-control when passing through doorways, small corridors, or complicated environments. This three-wheeled robot has three independent geared DC motors attached to the omnidirectional wheels that provide 3 DOF to the motion system. Each wheel has the same distance, R, from its center to the center of the mobile robot. The APR has a triangular contact area with the ground due to the three wheels used in the motion system.

The mobile robot has a thin body and an upper part mainly designed for human interaction. The head of the mobile robot has a multi-touch panoramic screen and two arms each with one degree of freedom in order to move them forward and backward. The chest and shoulders of the APR are located approximately at 1.3 m height, which is slightly lower than the average for human shoulders. The shoulders of the APR enclose two DC geared motors which are connected to two soft arms with a 35 cm separation in between. The arms are 55 cm long for esthetical reasons and can be used as a support by elderly people when walking or can be used for basic gesture interaction. The arms are periodically moved during a forward displacement in order to replicate the natural movements performed by humans when walking. Table 1 summarizes the main physical features of the two mobile robots used in this paper.

**Figure 3:**
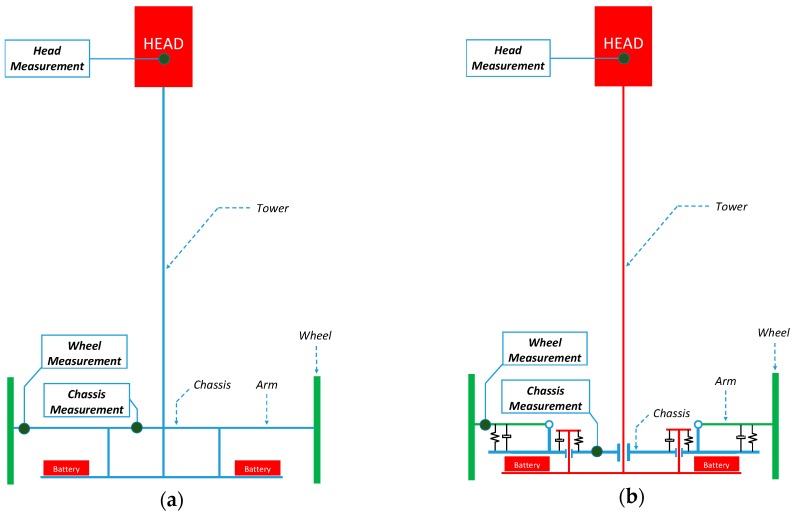
Structural diagram of the (**a**) APR-1 prototype and the (**b**) APR-2 prototype. The dark green dots depict the location of the measurement points: wheel, chassis, and head.

**Figure 4:**
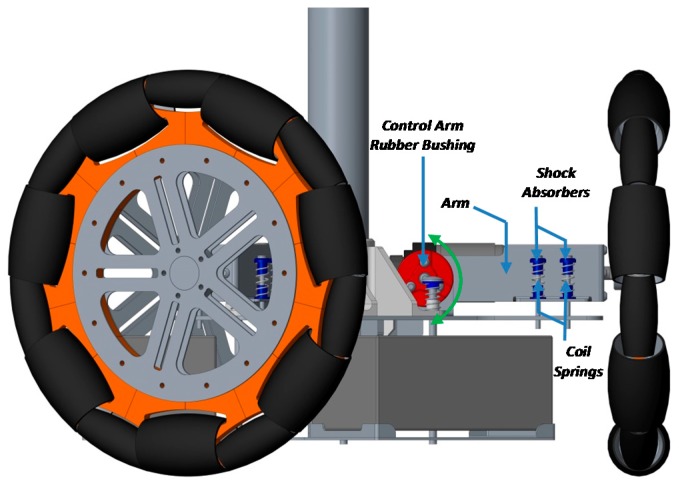
CAD design of the passive suspension system of the APR-2.

**Figure 5:**
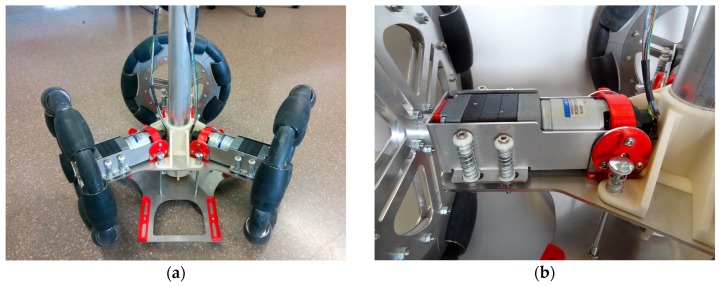
Image of the passive suspension system of the APR-2; (**a**) overview, (**b**) arm detail.

Figure 3 shows the structural diagram of the two mobile robot prototypes used in this paper. The chassis in the design of the APR-2 prototype (Figure 3b) improves the wheels and tower joints. The improved joints are based on a damped arm and a joint between the tower and the chassis, allowing the tower to balance and improve its stability as the pivot point is positioned above the tower’s center of gravity. Figure 4 shows the CAD design of the suspension system. Finally, Figure 5 shows an overview of the implemented suspension system (Figure 5a), and a close-up image of a single arm of the base (Figure 5b). The red parts of the APR-2 prototype are made of a flexible 3D printing filament. This prototype also includes two sets of spring-based connections; the first one is located between the motor’s and batteries’ structures; and the second one is found between the mobile robot batteries’ and the central tower’s structures. 

### 2.2. Profile Wheel Vibration

The most common holonomic motion system for robots is based on the use of three or four omnidirectional wheels. The operational function of the different types of omnidirectional wheels is based on providing traction in the natural direction towards the motor axis, and using numerous passive rollers (where the axes are at the tangent of the wheel circumference) that can slide in the direction of the motor axis. This implementation allows the wheel to spin as well as perform perpendicular displacements from the wheel’s forward trajectory, allowing the robot to move in any direction.

**Figure 6:**
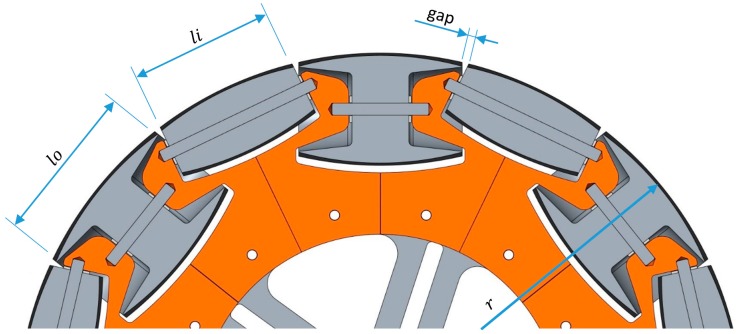
CAD section showing the alternate use of the two passive roller types and the gap between them.

The common motion system implemented in the APR is based on the use of three omnidirectional wheels shifted 120° and is composed of passive rollers placed along the periphery of the main wheel. Figure 6 shows the design of one omnidirectional wheel based on the use of alternating passive rollers with different sizes and shapes in order to minimize the gap between the rollers. Table 2 summarizes the main mechanical features of the wheels. In this case, the theoretical contact points between the wheel and the ground are aligned, but the gap between rollers and some manufacturing defects cause horizontal and vertical vibrations.

Equation (1) describes the analytical expression used to obtain the *gap* or distance between the rollers depending on the mechanical wheel features:

(1) $$gap=2\cdot r\cdot \sin\left(\frac{2\cdot \pi \cdot r-ni\left(2\cdot \sin^{-1}\left(\frac{li}{2\cdot r}\right)\cdot r\right)-no\left(2\cdot \sin^{-1}\left(\frac{lo}{2\cdot r}\right)\cdot r\right)}{2\cdot r}\right),$$

where *ni* and *no* are the number of the inside and outside passive rollers, *li* and *lo* are the length of the inside and outside passive rollers, and *r* is the radius of the wheel.

Equations (2) and (3) describe the analytical expression used to obtain the vertical displacement profile, *d*, of the center of the wheel when the gap between the passive rollers are in contact with the ground.

(2) $$d(\alpha )=\frac{\left|r\cdot \left(\sin\left(\frac{3\cdot \pi }{2}-\sin^{-1}\left(\frac{gap}{2\cdot r}\right)\right)+\frac{\cos\left(\frac{3\cdot \pi }{2}-\sin^{-1}\left(\frac{gap}{2\cdot r}\right)\right)}{\tan\left(\left(\frac{3\cdot \pi }{2}-\sin^{-1}\left(\frac{gap}{2\cdot r}\right)\right)+\alpha \right)}\right)\right|}{\sqrt{{\left(\frac{-1}{\tan\left(\left(\frac{3\cdot \pi }{2}-\sin^{-1}\left(\frac{gap}{2\cdot r}\right)\right)+\alpha \right)}\right)}^{2}+{\left(-1\right)}^{2}}}\;\to \;\alpha =0:to:\sin^{-1}\left(\frac{gap}{2\cdot r}\right),$$

(3) $$d_{max}=r-\sqrt{r^{2}-{\left(\frac{gap}{2}\right)}^{2}},$$

where α is the angle that forms the *gap*.

**Figure 7:**
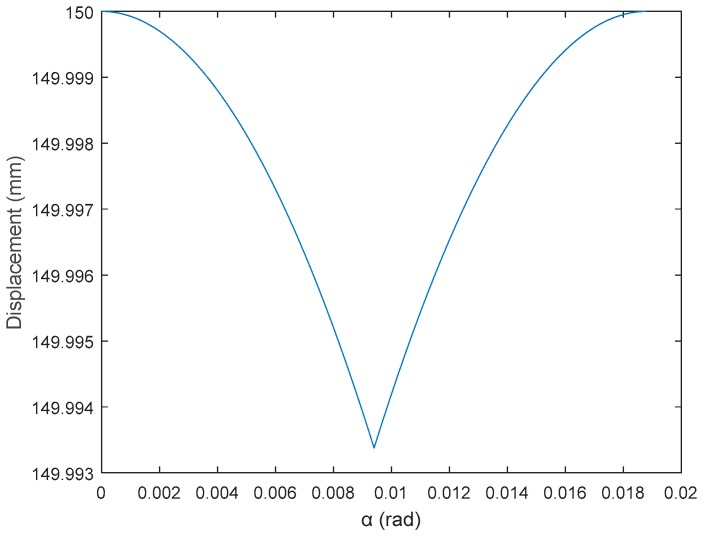
Profile of the vertical displacement of the wheel’s center found across the gap.

Figure 7 shows the vertical displacement profile’s typical shape, *d*, that appears in the center of the omnidirectional wheel due to the gap between the rollers. This displacement profile has a sharp transition that causes strong vertical vibrations. The maximum theoretical amplitude or displacement is 0.006 mm but in the practice this value is also affected by the weight of the mobile robot that crushes asymmetrically the soft cover of the passive rollers.

### 2.3. Vibration Measurement

The vibrations measurement is carried out by a LIS3DSH accelerometer manufactured by STMicroelectronics (Rennes, Switzerland). The LIS3DSH is a low-power high-performance three-axis linear accelerometer of compact size (3 × 3 × 1 mm), the dynamic measurement range is selectable between ±2 to ±16 g, the sensitivity is between 0.06 to 0.73 mg/digit depending on the range, and it has a maximum sampling rate of 1.6 kHz. 

**Figure 8:**
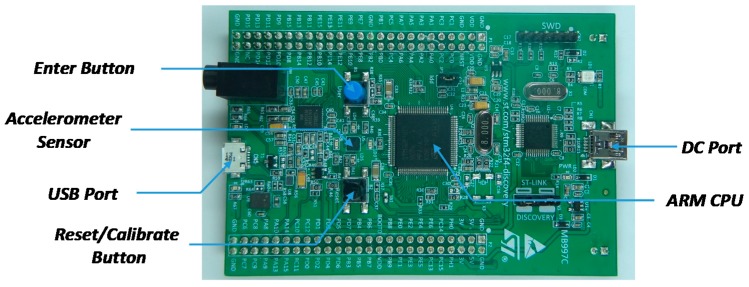
Data acquisition system based on the STM32F4-Discovery board.

The complete data acquisition system is based on the STM32F4-Discovery board from STMicroelectronics that includes on the same board the LIS3DSH accelerometer sensor and a STM32F407VGT6 32-bit high-performance microcontroller based on the ARM Cortex™-M4 processor developed by ARM (Cambridge, UK), and which is also manufactured by STMicroelectronics (Figure 8). This acquisition system has an USB 2.0 on the go (OTG) interface for connectivity, a reset pushbutton, and a user configurable pushbutton used for data or experiment triggering.

The microcontroller uses an internal timer to read the raw accelerometer data using a Serial Port Interface (SPI). The maximum sampling rate is 1.6 kHz and the raw accelerometer data is stored in the microcontroller’s RAM and then transferred as a text file to a USB flash-disk memory connected to the USB 2.0 OTG interface. The microcontroller is able to store up to 12,424 samples of the three-axis accelerometer, which means 7.765 s at the maximum sampling rate supported by the accelerometer. 

The measurements have been carried out by configuring the accelerometers with a dynamic range of ±4 g and a sensitivity of 0.12 mg/digit. This range has been obtained by a trial and error procedure in order to avoid any saturation in the raw data obtained from the accelerometers. The standard deviations of the sensor for the *X*, *Y*, and *Z* axes at zero speed were 0.0132 g, 0.0181 g, and 0.0144 g, respectively.

## 3. Measurements

**Figure 9:**
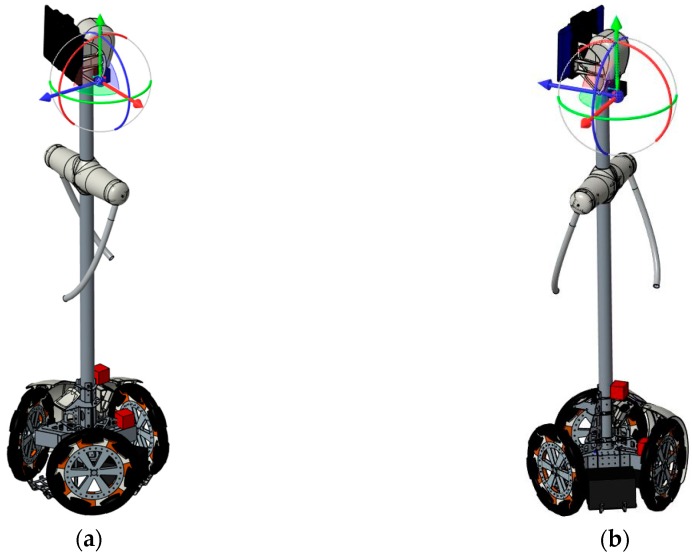
Mobile robot APR-1 with a red box representing the location of the accelerometers: front (**a**) and side (**b**) view. The longitudinal, normal, and side axes of the measurements are depicted with a blue, green, and red arrow, respectively.

The vibration measurement has been performed by attaching the accelerometers to one wheel, to the chassis, and to the top of the mobile robot (see Figure 3 and Figure 9 for reference). The accelerometers’ axes orientation is the same as the reference axes plotted in Figure 9, where the *Z* axis corresponds to the normal vector, the *X* axis to the longitudinal vector, and the *Y* axis corresponds to the side vector.

Table 3, Table 4 and Table 5 show the raw data obtained by the accelerometers located on both robots along the longitudinal, side, and normal axes, respectively. The maximum speed of the two mobile robots is 100 cm/s. The operational speed range is in an array from 19 and 66 cm/s hence the data provided in the tables depicts these two extreme velocity cases. The gathered data from the APR-1 (without suspension) is shown in blue; the gathered data from the APR-2 (with passive suspension) is shown in orange.

Table 3 and Table 4 show the vibrations’ dynamic evolution measured in both the wheels and chassis of the two mobile robots. The dynamic evolution is similar even though the APR-2 prototype (with a passive suspension) reduces the overall vibrations’ amplitude in the normal axis, hence a reduction in the transmission of the vibrations to the upper part of the mobile robot is expected. Table 3 and Table 4 also show the presence of some acceleration peaks in the APR-2 prototype caused by small irregularities on the ground, although these peaks are not directly transmitted to the head of the mobile robots (see Table 5).

Table 5 shows the vibrations’ dynamic evolution measured at the head of the two mobile robots at two velocities. In this case, the passive suspension system has reduced the overall amplitude and peaks of the vibrations measured in all axes. The maximum amplitude of the vibrations in the APR-1 prototype (without suspension) was raised from 0.45 g for a forward velocity of 19 cm/s to 3.97 g for a forward velocity of 66 cm/s (~8.8 times). The vibrations’ maximum amplitude measured in the APR-2 prototype (with passive suspension) was raised from 0.17 to 0.40 g in the same velocity range (~2.3 times). Therefore, the vibrations’ maximum amplitude in the head of the APR-2 prototype was lower than the minimum vibrations measured in the APR-1 prototype. Additionally, the measurements over the longitudinal axis also showed a smooth undamped effect caused by the passive suspension when accelerating and decelerating the APR-2 prototype.

**Figure 10:**
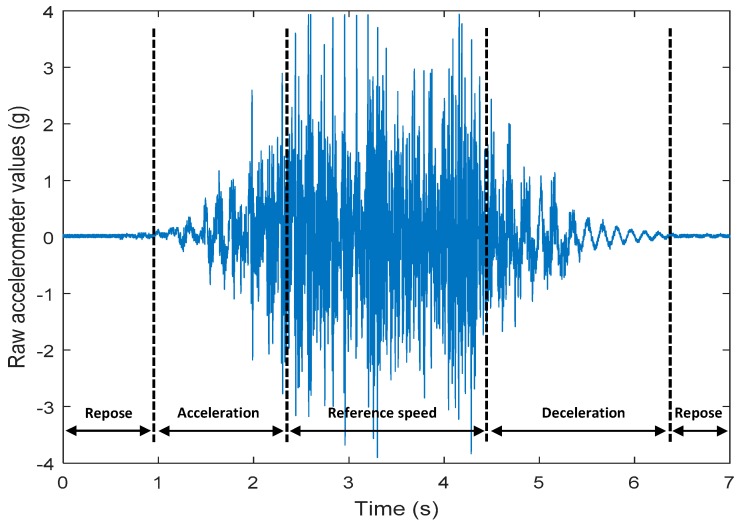
Raw accelerometer data obtained at the head of the APR-1 prototype when the forward speed was fixed at 66 cm/s.

**Figure 11:**
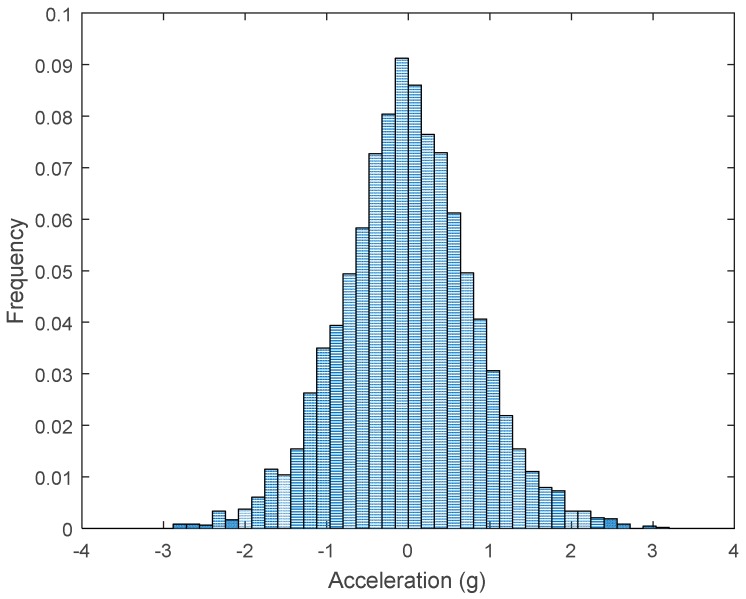
Histogram of the side accelerations measured at the head of the APR-1 prototype when moving at a constant velocity of 55 cm/s during a forward displacement.

The raw data measured during the displacements has been manually divided in four different states: repose, initial acceleration, constant speed, and deceleration (Figure 10). The data gathered by the accelerometers while the robot is going forward at a constant speed (reference speed) has been selected to create a histogram of the accelerations’ amplitude measured during the displacement. As an example, Figure 11 shows the histogram of the side acceleration measured at the head of APR-1 when moving forward at a reference velocity of 55 cm/s. Figure 11 shows a typical Gaussian distribution with a standard deviation of 0.80 g. The value of the standard deviation will be used in this paper to compare the overall vibrations measured in both mobile robots.

For the sake of comparison, Table 6, Table 7 and Table 8 summarize the vibration pattern histograms obtained from each mobile robot when moving forward at two different constant velocities (23 and 66 cm/s). These histograms also represent a typical normal distribution. Note that, in some cases, the Gaussian distribution is not zero centered due to small misalignments between the accelerometer axes and gravity. 

Table 9 compares the evolution of the standard vibration deviation measured at different velocities in the wheel, chassis, and head of the APR-1 and APR-2 mobile robot prototypes. The results show that the vibrations on the APR-1 (without suspension) have a clear tendency to increase proportionally with the velocity. Alternatively, the vibrations measured at the head of the APR-2 (with passive suspension) are almost constant with a standard deviation of 0.80 g at the maximum speed. This is because in the first prototype the wheels’ vibrations are directly transmitted and amplified due to the tower-typed structure, reaching a maximum acceleration peak of almost 4 g at the head when moving forward at a constant speed of 55.2 cm/s. The measurements also show that the longitudinal and side measures at the chassis of the APR-1 are similar while there is a considerable variation between them and the normal axis; this is mainly caused by the edge of the rollers hitting the ground during their transition, shaking the structure along the normal axis.

The acceleration data gathered at the head of the APR-2 shows that the suspension system allows the central structure to balance itself in a controlled way when the robot is moving at a constant velocity. The measures obtained at the wheel of the APR-2 prototype show that the addition of flexible shock absorbers and spring based suspensions are an effective method to minimize and stabilize the wheels’ vibrations which are almost constant and not transmitted to the tower. The rigid APR-1 prototype cannot absorb the vibrations originating in the wheels which are directly transmitted to the chassis and the tower of the mobile robot.

**Figure 12:**
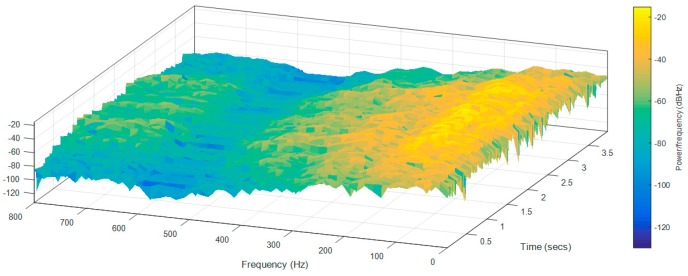
Frequency spectra of the accelerations measured over the longitudinal axis at the head of the APR-1 prototype (without suspension).

**Figure 13:**
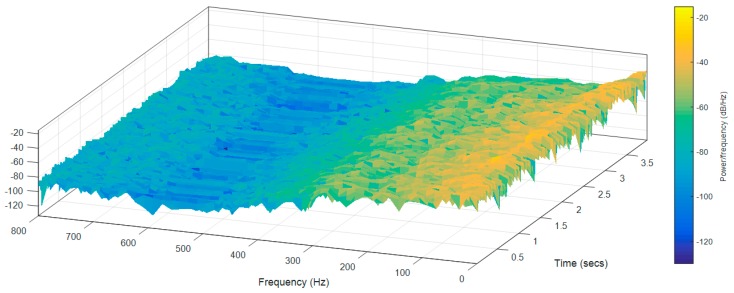
Frequency spectra of the accelerations measured over the longitudinal axis at the head of the APR-2 prototype (with passive suspension).

Finally, Figure 12 and Figure 13 show the frequency spectra of the accelerations measured over the longitudinal axis at the head of the two mobile robot prototypes in the case of moving forward at a constant speed of 66 cm/s (Table 9—Longitudinal and Head data). Figure 12 shows that in the case of the APR-1 prototype (without suspension), the highest peak frequencies are in a range from 10 to 300 Hz, while Figure 13 shows that in the case of the APR-2 prototype (with passive suspension), these frequencies have overall smaller amplitudes. These spectra differences go along with the results shown in Table 9 based on the computation of the standard deviation of the vibrations measured in the mobile robots.

## 4. Conclusions

This paper presents the vibration pattern measurements obtained from two tower-typed mobile robots based on a holonomic motion system. The mobile robots are both configured as Assistant Personal Robots (APR). The first implemented prototype (APR-1) includes a rigid structure whereas the second implemented prototype (APR-2) includes a passive suspension system. The design of the APR-2 prototype includes flexible joints between the wheels and the chassis and between the chassis and the tower, and also a damped arm between the chassis and the tower. This design improves the stability as the pivot point is positioned above the center of gravity of the tower. The vibrations have been measured in one wheel, the chassis, and the head of the tower-type mobile robots. In both cases the omnidirectional wheels’ gap is the main vibration source. The results have shown that the vibration amplitude measured on both mobile robots when going forward at a constant speed have a typical Gaussian distribution that can be modelled with the value of the standard deviation, and similar results are shown for the vibrations measured in the wheels and in the chassis of the two mobile robots, as they use the same wheel design. Similarly, the results have shown a large reduction of the vibration standard deviation measured in the head of the mobile robot without suspension (1.3 g) and with the passive suspension system (0.08 g). These results represent a huge vibration reduction (1/16) and a huge reduction of associated problems, such as the electronic devices’ mechanical stress located at the head of the mobile robots.

## Figures and Tables

**Table 1:** Physical specifications of the two APR prototypes.

	Height (cm)	Width (cm)	Weight (kg)	Suspension	Screen (Inches)
**APR-1**	164	48	34.5	No	7
**APR-2**	168	54	38.3	Yes	13

**Table 2:** Main mechanical features of the omnidirectional wheels.

Diameter (cm)	Width (cm)	Outer Rollers Specifications	Inner Rollers Specifications	Gap (mm)	Maximum Vertical Displacement ^1^ (mm)
300	44.48	7 units Ø_max_ 44.48 mm Length 67.5 mm	7 units Ø_max_ 26.52 mm Length 60.5 mm	2.82	0.0066

^1^ Maximum distance of the typical shape of the vertical displacement profile.

**Table 3:** Vibration representation measured in the wheel of the APR-1 and APR-2 prototypes.

	Forward Velocity (Wheel Measurements)	
	19 (cm/s)	66 (cm/s)
Longitudinal	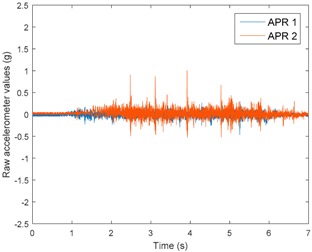	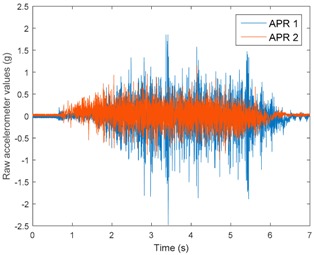
Side	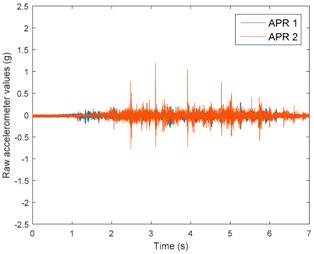	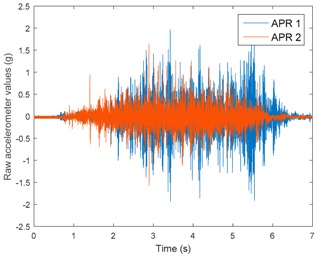
Normal	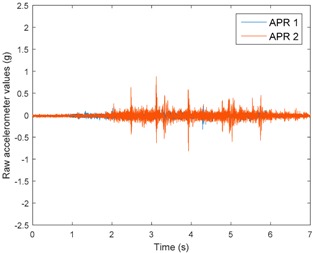	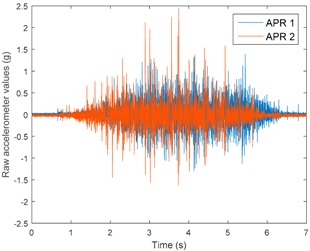

**Table 4:** Vibration representation measured in the chassis of the APR-1 and APR-2 prototypes.

	Forward Velocity (Chassis Measurements)	
	19 (cm/s)	66 (cm/s)
Longitudinal	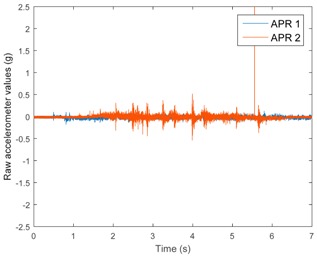	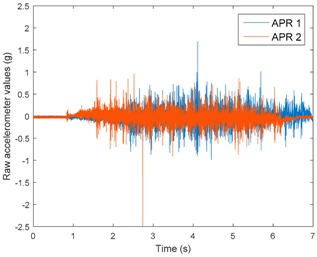
Side	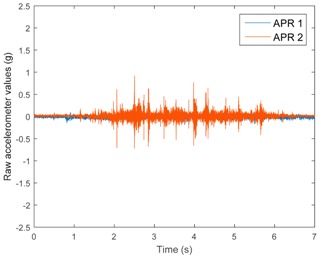	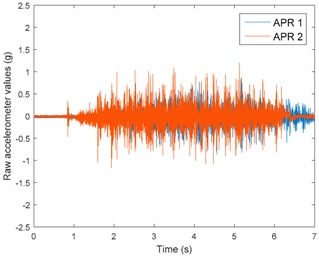
Normal	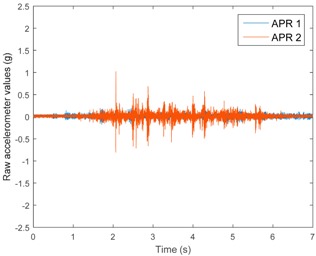	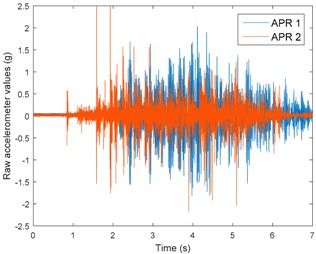

**Table 5:** Vibration representation measured at the head of the APR-1 and APR-2 prototypes.

	Forward Velocity (Head Measurements)	
	19 (cm/s)	66 (cm/s)
Longitudinal	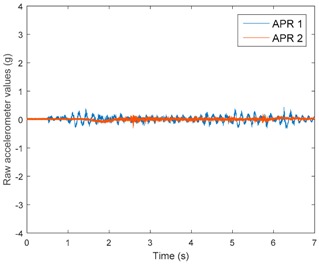	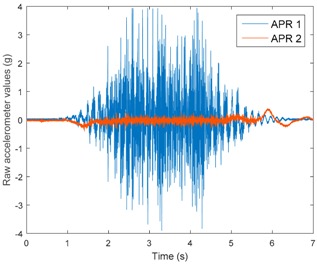
Side	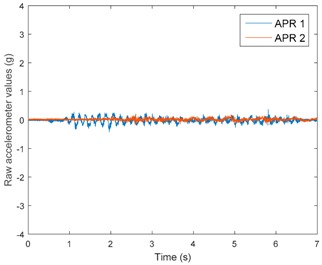	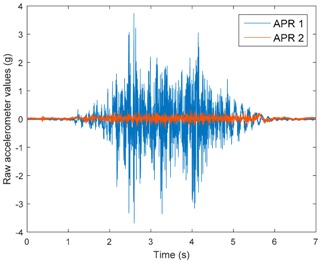
Normal	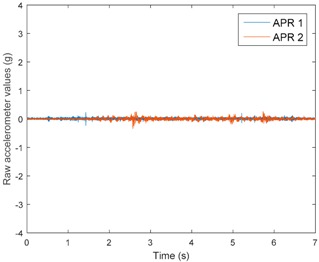	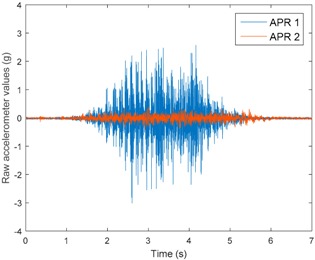

**Table 6:** Vibration pattern histograms measured in the wheel of the two APR prototypes.

	Forward Velocity (Wheel Measurement)	
	23 (cm/s)	66 (cm/s)
Longitudinal	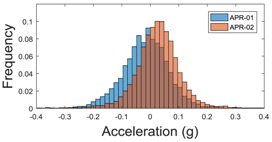	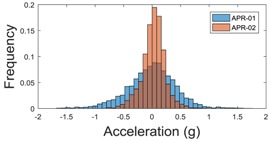
Side	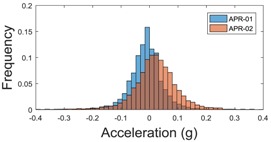	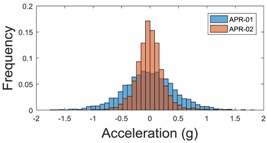
Normal	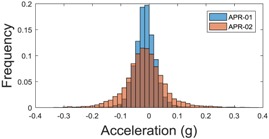	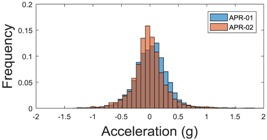

**Table 7:** Vibration pattern histograms measured in the chassis of the two APR prototypes.

	Forward Velocity (Chassis Measurement)	
	23 (cm/s)	66 (cm/s)
Longitudinal	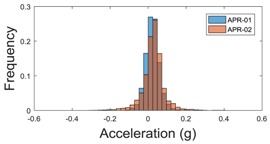	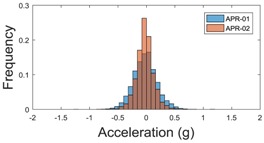
Side	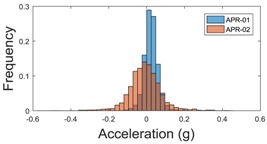	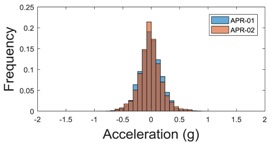
Normal	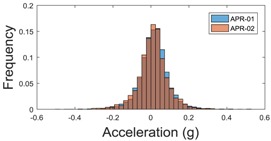	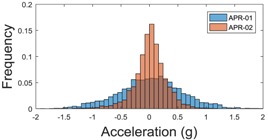

**Table 8:** Vibration pattern histogram measured in the head of the two APR prototypes.

	Forward Velocity (Head Measurement)	
	23 cm/s	66 (cm/s)
Longitudinal	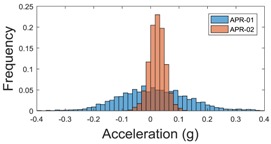	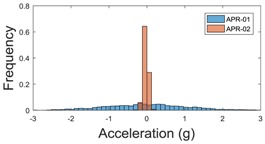
Side	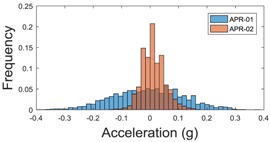	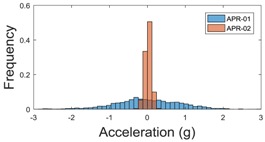
Normal	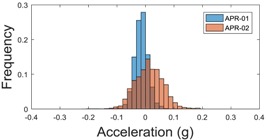	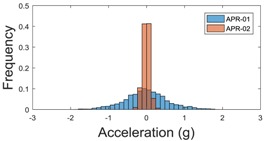

**Table 9:** Comparison of the vibrations’ standard deviation measured in the APR-1 and APR-2 prototypes.

	Standard Deviation of the Acceleration Measured at the:		
	Wheel	Chassis	Head
Longitudinal	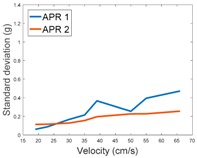	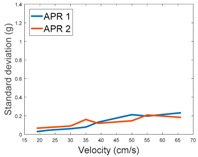	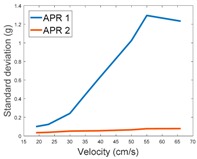
Side	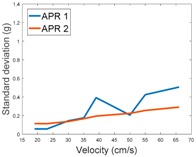	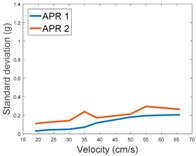	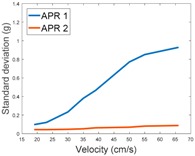
Normal